# Digital health interventions for childhood obesity: An umbrella review

**DOI:** 10.1177/20552076261415914

**Published:** 2026-04-01

**Authors:** Edlin Glane Mathias, Siva N, Elstin Anburaj S, Vaishnavi Naik, Baby S Nayak

**Affiliations:** 1 Department of Health Technology and Informatics, Prasanna School of Public Health, 76804 Manipal Academy of Higher Education, MAHE, Udupi district, Karnataka, India; 2 Centre for Evidence-informed Decision making, Prasanna School of Public Health, 76804 Manipal Academy of Higher Education, MAHE, Manipal, Udupi district, Karnataka, India; 3 Department of Child Health Nursing, SUM Nursing College, 78942 Siksha ‘O’ Anusandhan University, Bhubaneshwar, Odisha, India; 4 Department of Child Health Nursing, Manipal College of Nursing, 76804 Manipal Academy of Higher Education, MAHE, Udupi district, Karnataka, India

**Keywords:** Childhood obesity, BMI, digital health, mHealth, eHealth, behavior change, public health

## Abstract

**Introduction:**

Childhood obesity has become a critical global health issue, raising concerns for public health systems. Early intervention is essential to curb its long-term impact.

**Objective:**

This umbrella review aims to assess the effectiveness of digital health interventions in controlling and managing obesity among children and adolescents.

**Methods:**

Following Cochrane methodology, comprehensive searches were conducted in PubMed, CINAHL, Cochrane Library, Web of Science, Embase, and ProQuest Health and Medical Library, EPISTEMONIKOS, and the Joanna Briggs Institute Database. Eligible reviews included studies targeting individuals aged ≤18 years children who were overweight or obese based on BMI-for-age percentiles or *z*-scores. Interventions explored include mobile health (mHealth), electronic health (eHealth), mobile apps, SMS, telehealth, and wearable technologies. No restrictions were placed on comparators. Primary outcomes included BMI, BMI *z*-scores, BMI-for-age percentiles, and related anthropometric indicators. Screening was independently conducted by two reviewers using Rayyan software. The methodological quality of each review was assessed using the AMSTAR-2 tool, with findings synthesized narratively.

**Results:**

Out of 242 records screened, 27 systematic reviews were included. Ten reviews examined mHealth interventions, four explored eHealth or web-based strategies, while others focused on telehealth, wearable devices, or multicomponent approaches. A combination of mobile and SMS-based tools consistently demonstrated greater potential in supporting BMI reduction and promoting behavioral change.

**Conclusion:**

Mobile and SMS-based digital interventions show promise in managing childhood obesity. Their integration into public health strategies could enhance the effectiveness of weight management efforts among children and adolescents.

## Introduction

Childhood obesity has emerged as a critical global health concern, with its prevalence growing at an alarming rate. In 2022, an estimated 37 million children under the age of five were overweight, and over 390 million children and adolescents aged 5–19 years were classified as overweight or children with obesity, including 160 million with obesity.^1,2^ This represents a significant increase from 8% in 1990 to 20% in 2022, with similar trends observed among both boys (21%) and girls (19%).^1^ Obesity is a chronic condition characterized by excessive fat deposits that can negatively impact the health of individuals. It is typically diagnosed with Body Mass Index (BMI) measurements, which correspond to the height and weight of individuals. In children and adolescents, overweight and obesity are not assessed using fixed BMI thresholds as in adults but rather using BMI-for-age percentiles or BMI *z*-scores, which account for age- and sex-specific growth patterns. For example, the World Health Organization defines overweight in children as a BMI-for-age greater than +1 standard deviation (SD) (equivalent to the 85th percentile) and obesity as greater than +2 SDs (approximately the 97th percentile). The Centers for Disease Control and Prevention similarly define overweight as a BMI at or above the 85th percentile and below the 95th percentile and obesity as at or above the 95th percentile for children of the same age and sex.

Childhood obesity is linked to numerous health complications, including metabolic, cardiovascular, psychological, orthopedic, neurological, hepatic, pulmonary, and renal disorders.^3^ It can also affect bone health, reproduction, and the risk of certain cancers. Moreover, obesity impacts daily activities, such as sleep and mobility, and significantly reduces the quality of life.^1^ Managing childhood obesity requires a multidisciplinary approach, combining dietary management, increased physical activity, reduced sedentary behaviors, pharmacotherapy, and, in severe cases, bariatric surgery. Factors contributing to childhood obesity include unhealthy diets, physical inactivity, genetics, and socioeconomic status..^4^ Being overweight during childhood often results in long-term comorbidities, extending into adulthood, and poses a significant risk for chronic diseases later in life.^2^ In this review, “controlling obesity” is broadly defined to include efforts aimed at the treatment or reduction of existing overweight and obesity, prevention of further weight gain, and behavioral modifications that support a healthy weight. This encompasses changes in body mass index (BMI) or BMI *z*-scores, as well as improvements in physical activity, dietary habits, and other lifestyle-related outcomes associated with childhood obesity prevention and management.

The COVID-19 pandemic augmented the implementation of digital health technologies, offering new opportunities to deliver health care services remotely and also at a larger scale.^5,6^ While traditional obesity interventions such as in-person counseling, structured physical activity programs, school-based initiatives, and family-based approaches have proven effective, they face challenges in accessibility, commitment, and long-term sustainability, especially among children with evolving needs and behaviors.^7,8^ Digital health interventions (DHIs) have emerged as promising tools for obesity prevention and management in children.^9,10^ DHIs are defined as the use of information and communication technologies, including mobile health (mHealth), electronic health (eHealth), web-based platforms, mobile applications, text messaging services (SMS), smartphone-based programs, computer- and game-based interventions to deliver health promotion, prevention, or treatment programs aimed at reducing or managing childhood obesity. These interventions may be delivered through various digital modalities such as smartphones, tablets, computers, or wearables, with or without human support.

Addressing childhood obesity requires collaborative efforts involving governments, healthcare providers, schools, and families. Educational initiatives promoting nutrition and physical activity, alongside supportive environments that encourage healthy behaviors, are critical in mitigating this epidemic. Despite the growing evidence on DHIs, the evidence remains fragmented across systematic reviews with varying scopes, methodologies, and quality. This umbrella review aims to synthesize findings from systematic reviews appraising the effectiveness of DHIs in preventing and managing obesity among children and adolescents (≤18 years). By mapping the types of interventions, delivery contexts, and outcomes, we aim to inform the stakeholders, such as researchers, clinicians, and policymakers, on the design and implementation of effective, equitable, and sustainable digital approaches for the prevention and management of childhood obesity.

## Review question

- What digital health interventions are available for controlling and managing obesity among children and adolescents?
- How do these digital health interventions influence obesity management and prevention outcomes in children and adolescents?

## Methods

The Cochrane methodology for overviews was adopted in conducting this review.^10^ This approach systematically maps themes across studies and synthesizes findings to provide a thorough understanding of available interventions to control obesity among children. The steps of the review were guided by the PRISMA (Preferred Reporting Items for Systematic Reviews and Meta-Analyses) guidelines.^11^ This review protocol is registered with the International Prospective Register of Systematic Reviews (PROSPERO- CRD42024560675).

## Eligibility criteria

Based on the Population, Intervention, Context, and Outcome (PICO) framework, the inclusion and exclusion criteria were formulated.

**Population (P):** This review included children and adolescents aged 18 years and below who were overweight or obese, as defined by the authors in the included studies. Children and adolescents who were diagnosed with chronic eating disorders and psychological illnesses were excluded.

**Intervention (I):** In this review, digital health interventions (DHIs) refer to using digital technologies to support the prevention and management of obesity. This includes interventions targeting weight reduction, prevention of weight gain, or improvement of obesity-related behaviors such as diet, physical activity, and screen time. The included interventions may vary in scope and delivery. For this review, DHIs focusing on diet, physical activity, behavioral modification, or a combination of these aimed at weight management in children and adolescents (≤18 years) are included.

We included mobile health (mHealth), which involves using mobile and wireless technologies, such as smartphones, mobile applications, text messaging (SMS), and wearable devices, to deliver health services and promote behavior change. Electronic health (eHealth) encompasses a broader range of internet- and computer-based platforms, including web portals, telehealth, and software applications used in health care delivery. The reviews that included pharmacological interventions to control obesity were not included. Reviews focusing solely on the feasibility or acceptance levels of the applied technology-based interventions and interventions provided through social media and online education platforms were not included. The included digital health interventions may vary in scope and delivery mode. They encompass individual-level strategies such as mobile applications for self-monitoring, goal setting, or behavior tracking; family-based approaches involving parent-child digital education or behavior change programs; and broader public health-oriented interventions delivered at the school or community level through eHealth platforms. This range reflects the diversity in how digital tools are used to address obesity prevention and management among children and adolescents. For clarity and consistency, we use the term digital health interventions to collectively refer to both mHealth and eHealth strategies included in the scope of this review.

**Comparator:** In this review, we did not attempt to compare digital health interventions with any specific intervention. We included systematic reviews where DHIs were evaluated against a variety of comparators, including standard care or non-digital interventions. Any form of conventional care was considered acceptable as a comparator, allowing for a wide synthesis of evidence across various study designs.

**Context (C):** Systematic reviews conducted to control and manage obesity among children globally.

**Outcome:** The primary outcomes of this umbrella review were anthropometric indicators of adiposity, including body mass index (BMI), BMI *z*-scores, BMI percentiles, and waist circumference. If they reported at least one of these measures as a significant outcome, they were included. Secondary outcomes, such as physical activity levels, dietary habits, and screen time, included behavioral indicators such as physical activity levels. Outcome data were extracted as reported by the original review authors, based on their definitions and measurement approaches.

**Time:** Systematic reviews available in English till March 31, 2025.

**Study designs:** This review includes Systematic reviews of RCTS as well as of other quantitative studies. Primary articles, scoping reviews, qualitative reviews, editorials, conference proceedings, and systematic reviews of qualitative studies were excluded.

## Information sources

A broad and comprehensive search strategy was devised to identify existing systematic reviews relevant to digital health interventions for obesity among children and adolescents. The strategy included the selection of appropriate keywords from the Medical Subject Headings (MeSH) library, main headings, text words, and previous relevant reviews.

The following electronic databases were searched: PubMed (NCBI), CINAHL (EBSCO ultimate), Cochrane Library (Wiley), Web of Science (Clarivate), Embase (Elsevier), ProQuest Medical Library, EPISTEMONIKOS, and the Joanna Briggs Institute Database. The initial search strategy was developed for PubMed and subsequently adapted for the remaining databases. Database-specific modification done for each database. The Peer Review of Electronic Search Strategies (PRESS) checklist was utilized to validate the searches that were constructed.^12^ Conference abstracts and editorial commentaries were excluded, as our focus was on peer-reviewed systematic reviews with sufficient methodological detail. Reference lists of all the included reviews were hand-searched to identify additional eligible reviews. The complete PubMed search strategy is provided in Appendix File 1.

## Selection of the studies

To initiate the study selection, the search data from the multiple databases were imported into the Rayyan software.^13^ Deduplications were removed, and studies were sorted for further screening. A two-stage screening process was conducted following pre-defined inclusion and exclusion criteria. Two reviewers (MGE and NS) independently observed the titles and abstracts of the articles in the initial stages. Discrepancies in article selection were resolved through a consensus-building process. The entire text of the selected articles was examined independently by the same two reviewers in the second stage. Any disputes at this stage were resolved by consulting a third reviewer (BSN).

## Data extraction and management

The data relevant to our review was extracted using a pre-designed data extraction form in MS Excel. Initially, three studies were conducted to obtain sufficient information to make the data extraction sheet. Two reviewers (EGM & NS) were employed to extract data. The following data were collected from the systematic reviews: title, authors, year, total number of primary studies, number of participants, systematic review population, intervention, comparators, and primary and secondary outcomes.

## Methodological quality assessment

The revised AMSTAR-2 (Assessing the Methodological Quality of Systematic Reviews) tool was utilized to evaluate the quality of the included studies.^14^ According to the confidence in the evaluation results, the studies were rated as high confidence if there were no weaknesses or only one non-critical weakness. A high confidence level was given when more than one non-critical weakness was discovered. If there is a critical issue, with or without non-critical weaknesses, and if there is more than one crucial issue, with or without non-critical weaknesses, and without non-critical inadequacies, there is likely more than one critical issue. The methodology of each systematic review was examined independently by two reviewers (MGE and NS). Any disagreements were resolved through discussion until a consensus was reached.

## Data synthesis

A narrative synthesis approach was employed to summarize the findings, using tables and figures where appropriate to assist in the presentation and interpretation of the information.

## Results

**Figure 1.**
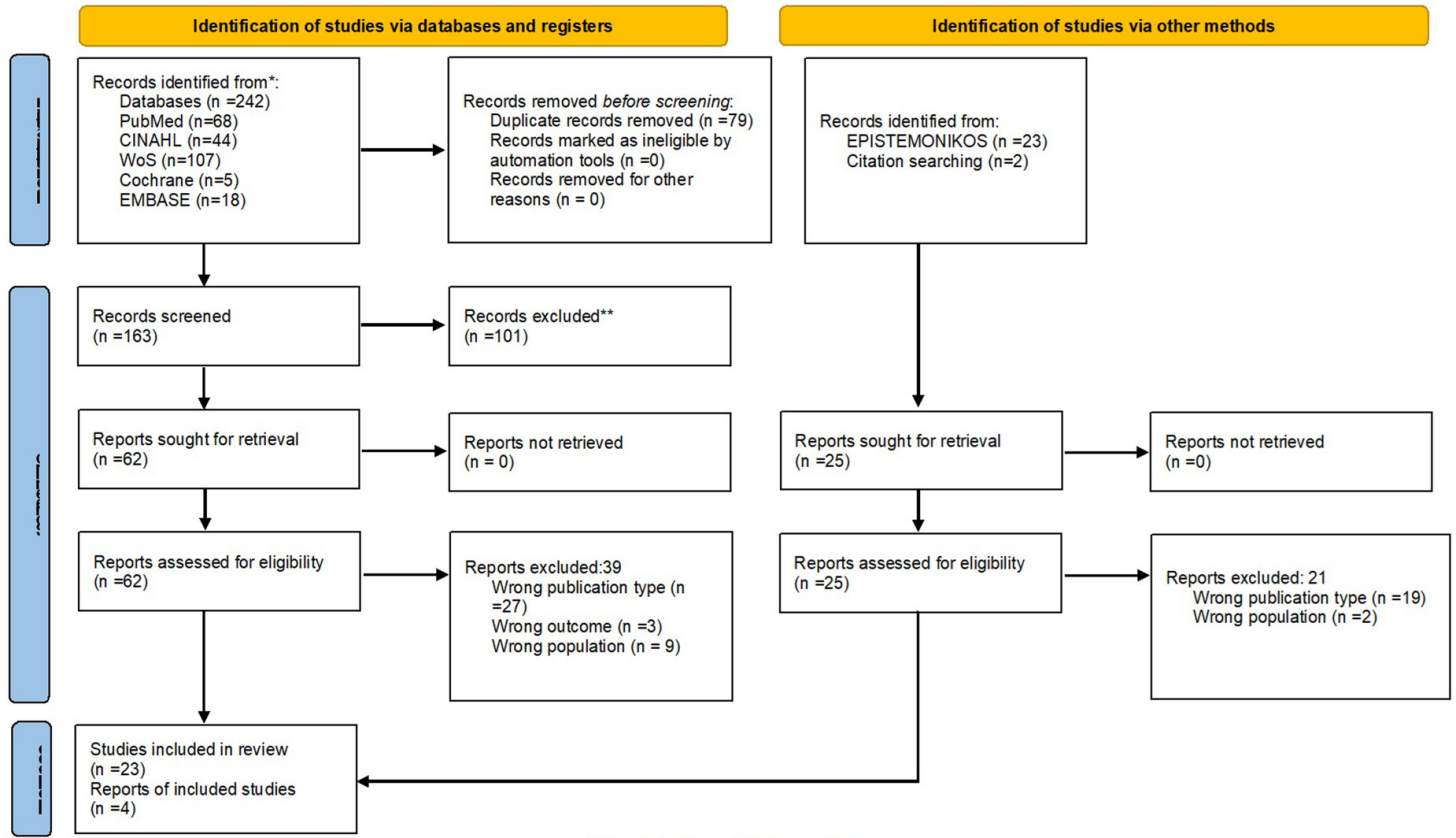
Study selection process.

The search generated 242 articles from the databases. The Rayyan software was used for deduplication. A total of 163 articles were presented for the title/abstract, and 62 were presented for the entire text. Due to not complying with the eligibility criteria, 39 studies were excluded: wrong publication type (*n* = 27), wrong outcome (*n* = 3), and wrong population (*n* = 9). A gray literature and citation search was also conducted, yielding 27 articles. After screening, 21 articles were excluded for the following reasons: incorrect publication type (*n* = 19) and incorrect population (*n* = 2). The PRISMA flow diagram depicts the screening process (Page et al., 2021) (Figure 1).

## Methodological quality of included systematic review

The methodological quality of the 27 included systematic reviews was evaluated using the AMSTAR 2 checklist, with ratings ranging from highly low to moderate. Fourteen reviews were rated as moderate quality, indicating multiple non-critical weaknesses but no critical flaws.^15–28^ One review was rated as low quality due to the presence of a critical flaw. These quality ratings have implications for the confidence we can place in the conclusions of this overview. Although the majority of reviews met basic methodological standards, the presence of several non-critical weaknesses in most reviews suggests that findings should be examined with caution. The limited number of high-quality reviews may reduce the overall effectiveness of the evidence base. Furthermore, although all reviews defined their research questions and inclusion criteria using the PICOS framework and outlined study selection plans, only two reviews^29,30^ used satisfactory methods to assess risk of bias in the included studies, and even these assessments were incompletely reported. In several instances, quality assessment of individual studies was either absent or based solely on the authors’ judgment, thereby limiting the reliability of the synthesized findings. The details of quality assessment of included systematic reviews are included in Appendix file 2.

## Summary of the included reviews

The characteristics of 27 distinct systems are presented in Table 1. These reviews included 473 primary studies describing 297 RCTs. Out of 27 reviews, 21 reviews were conducted in High-Income countries (HICs), two were conducted in Low-Income countries, and four were conducted in Upper- and Middle-Income Countries (UMICs). Ten reviews were conducted in the USA, three in China, two each in Korea, Iran, and Australia, one each in Canada, Finland, the UK, France, Italy, Greece, Taiwan, and Malaysia.

**Table 1.** Sample characteristics of the included studies.

S.No	Author name and year	Objective	Age	Gender	Sample size in overall studies	Number of studies included in the review	Type of studies included in review
1	Aminuddin NA and Azith NA. (2019) Malaysia	To evaluate the effectiveness of exercise games in reducing obesity among children	7–18 years	Not reported	311	6 studies	Four out of six articles included in this systematic review were RCTs, and another 2 articles were interventional studies
2	Antwi et al. (2013) New York	To identify the best available evidence on the effectiveness of web-based programs on the reduction of childhood obesity in school-aged children	4–18 years	Both boys and girls	883	12 studies	RCTs
3	Azevedo et al. (2021) UK	To examine the effectiveness of eHealth interventions for the treatment of children and adolescents with overweight or obesity	0–17 years	Mixed population	2352	19 in SR and 16 in meta-analysis	RCTs, cross-over trials
4	Bonvicini et al. (2022) Italy	To assess the efficacy and effectiveness of parents’ use of mobile health apps to prevent and treat childhood and adolescent obesity	1–18 years	Both male and female participants	Parents (2405), children (1990), dyads (3887), families (155), food pantry (n = 15) distributions including (N = 289) pantry clients	19 studies	RCTs, NRCTs
5	Wickham and Carbone (2015) USA	To assess and synthesize the literature on adolescent weight loss programs that utilize cell phones as an intervention component to reduce weight, as measured by body mass index or body mass index *z*-score	12–18 years	Both male and female adolescents	30–357	8 studies	RCTs and cohort
6	Chaplais et al. (2015) France	To provide a comparative evaluation of the effectiveness of using smartphones in the multidisciplinary treatment of child and adolescent overweight or obesity, with a specific interest in behavior change	7–17 years	Both male and female adolescents	190	2 studies	RCTs
7	Fowler et al. (2021) USA	To examine the efficacy of recent technology-based interventions on weight outcomes	1–18 years	Both males and females	27–2102	52 studies	RCTs
8	Hammersley et al. (2016) Australia	To review the evidence for BMI *z*-score improvements in eHealth overweight and obesity randomized controlled trials for children and adolescents, where parents or carers were an agent of change	Children and adolescents aged 0–18 years	Both male and female adolescents	1487 parent and child or adolescent dyads	8 studies	RCTs
9	Islam et al. (2020) Taiwan	To assess the efficacy of a mobile phone app intervention for reducing body weight and increasing physical activity among children and adults	Mean age of participants ranges from 12.7 to 44.9 years	Both male and female adolescents	1714	12 studies	RCTs
10	Kaakinen et al. (2017) Finland	To describe technology-based counseling interventions in weight and lifestyle management for obese or overweight children and adolescents	Children and adolescents below 18 years of age	Both male and female adolescents	8939	28 studies	RCTs, quasi-experimental, pre-post-test studies, interventional studies, post treatment study
11	Kepper et al. (2021) USA	To synthesize the latest evidence of health information technology used by healthcare professionals to address overweight/obesity among children and adolescents	2–18 years	Both male and female adolescents	34 to 2340	25 studies	RCTs, quasi-experimental studies, pre-post studies, randomized chart reviews, and single-arm studies.
12	Kouvari et al. (2022) Greece	To examine the effect of technology-based interventions on overweight and obesity treatment in children and adolescents.	Adolescents and children aged ≤18 years	Both boys and girls	582	9 studies	Controlled clinical trials
13	Lam et al. (2022) USA	To synthesize the evidence on the use of IoT-enabled technologies as an intervention for childhood obesity	Under the age of 18 years	Both boys and girls	3700	23 studies	RCTs, non-inferiority trial, non-randomized trials, cohort study, mixed methods study, intervention studies, pilot studies, evaluation studies, and system design studies
14	Langarizadeh et al. (2021) Iran	To appraise the potency of interventions based on mobile phone apps compared with other interventions to manage obesity and increase physical activity in populations of children and adolescents.	Children and adolescents aged 18 years or younger	Both male and female adolescents	978	9 studies	RCTs, quasi-experimental studies
15	Margetin et al. (2021) USA	The effect of telehealth weight management interventions compared to usual care on anthropometric outcomes in children and adolescents with overweight and obesity	Children aged 2–18 years	Both male and female adolescents	4071	13 studies	RCTs
16	Meidani et al. (2018) Iran	To investigate the role of phones (telephone and mobile phone) in interventions designed to control obesity in children under age six and to determine the features and effects of these interventions	Children aged 1 month to 6 years	Not reported	1460	5 studies	RCTs
17	Metzendorf et al. (2024) USA	To assess the effects of integrated smartphone applications for adolescents and adults with overweight or obesity	Adolescents’ mean age ranges from 12.6 to 16 years	Both boys and girls	254 adolescents	18 studies	RCTs
18	Park et al. (2021) Republic of Korea	To assess the effectiveness of child-centered ICT interventions on obesity-related outcomes	Aged <18 years	Both boys and girls	1259	10 studies	RCTs
19	Partridge et al. (2020) Australia	To determine the effectiveness of text message interventions in reducing BMI in adolescents and describe characteristics that are common to effective interventions.	10–19 years	Both boys and girls	767	8 studies	RCTs
20	Quelly et al. (2016) USA	To examine the impact of mobile app technology on obesity-related anthropometric, psychosocial, and behavioral outcomes in children and adolescents	9–19 years, with most studies focusing on 11–17-year-olds.	Both male and female	1149	9 studies	RCTs
21	Qiu et al. (2022) China	To investigate the effect of multiple eHealth-delivered lifestyle interventions on obesity-related anthropometric outcomes in children and adolescents	6–18 years, with a mean age of 12.38 years	Both male and female	64403	40 studies	RCTs
22	Shin et al. (2019) Korea	To evaluate the effectiveness of mobile phone intervention in promoting a healthy lifestyle among adolescents.	10–19 years	Both male and female adolescents	1472	11 studies	RCTs and non-RCTs
23	Smith et al. (2013) USA	To examine the effect of health IT (electronic health records [EHRs], telemedicine, text message or telephone support) on patient outcomes and care processes in pediatric obesity management	2–18 years	Both boys and girls	17 to 60,711	13 studies	RCTs, nonrandomized controlled trials, before-and-after studies, and cross-sectional studies
24	Turner et al. (2015) USA	To examine current use of mHealth technologies in the prevention or treatment of pediatric obesity to catalogue the types of technologies utilized and the impact of mHealth to improve obesity-related outcomes in youth	0–19 years	Mixed population	Not reported	41 studies	RCTs, pilot intervention studies, technology use and design studies, feasibility trials, and case reports.
25	Wang et al. (2022) China	To evaluate the effectiveness of wearable devices as physical activity interventions on obesity-related anthropometric outcomes in children and adolescents	Children and adolescents aged 6–18 years	Mixed population	3227	12 studies	RCTs
26	Yau et al. (2022) Canada	To examine the effectiveness of mobile apps aimed at preventing childhood obesity by promoting health behavior changes in diet, physical activity, or sedentary behavior in children aged 8–12 years.	Children aged 8–12 years	Both males and females	2477	13 studies	Quasi-experimental, RCTs
27	Wang et al. (2024) China	To analyze the effectiveness of mHealth app–based interventions in promoting PA and improving PF and identify potential moderators of the efficacy of mHealth app–based interventions in children and adolescents	Children and adolescents aged 3–18 years	Both males and females	5643	28 studies	RCTs

## Description of population

The primary research conducted in this overview of systematic reviews included children aged 0–18 years. The total number of participants included in the review was 1,20,772 (Table 1).

## Description of the included studies

The total number of studies in each review varied from 2 to 41. The interventions provided to prevent obesity varied throughout the review, where the interventions were delivered either in the laboratory, home, health center, community, participant homes, summer camps, outpatient clinics, or classroom. Most of the studies included web-based platforms to provide the intervention, while a few focused on eHealth interventions. Of 27 reviews, four reviews opted for web-based interventions to manage obesity; four reviews were on eHealth, 10 reviews focused on mHealth interventions, and one review had a combination of eHealth and mHealth interventions. Three reviews concentrate on the usage of telehealth technology, and three reviews included multicomponent interventions delivered through multiple technologies (mobile apps, text messages, phone sessions, face-to-face meetings, and web-based platforms). One review focused on wearable devices and behavioral strategies. The description of interventions is provided in Table 2.

**Table 2.** Description of intervention and outcomes.

S.No	Author (year)	Intervention type	Duration	Mode of delivery	Provider	Outcome metric (BMI/BMI *z*-score)	Key results (MD/SMD/WMD/95% CI/*P*-value)	Other findings
1	Aminuddin and Azith (2019), Malaysia	Web-based/Exercise games	Weeks to 6 months	Exercise games (individual/group)	Not reported	BMI	Reported reduction in BMI and adipose tissue; increased physical activity	Positive psychological impact; mixed reporting; one short 72-h study
2	Antwi et al. (2013), USA	Web-based programs	5–52 weeks	Web content, online journaling, email, discussion groups	Structured program with parental involvement	BMI, BMI *z*-score	Post-intervention BMI ∼34.07 (SD 6.57); one study ↓ BMI (*t* = -2.7, *P* < .01) and ↓ BMI *z*-score (*t* = -3.1, *P* < .01) at 9 months; 1 study ↓ BMI *z* at 16 weeks (F[5,60] = 5.11, *P* = .027)	4/8 interventions improved weight measures: varied follow-up (4 months–2 years)
3	Azevedo et al. (2021), UK	eHealth/mHealth	<12 months to 12 months	Telemedicine, SMS, mobile apps, wearables, active video games	Multidisciplinary HCP team	BMI (SMD)	SMD = 0.31 (95% CI 0.13 to 0.49), *P* < 0.001 (favoring eHealth)	Effect seen across ages <12 and 12–17; small effect size
4	Bonvicini et al. (2022), Italy	mHealth (parent-use apps)	4 weeks -1 year	Smartphone apps ± in-person/educational components	Healthcare providers/researchers	BMI *z*-score	One study: 6-month ΔBMIz intervention −0.23 (95% CI −0.33 to −0.13) vs standard care 0.01 (95% CI −0.1 to 0.11), *P* = .002	Mixed results across studies; several showed dietary behavior changes
5	Wickham and Carbone (2015), USA	Behavioral/lifestyle programs supported by mHealth	4 days -24 months	SMS, telephone coaching, email, mobile motion sensors, photo food logs	Health professionals/researchers	BMI *z*-score	↓ BMI *z*-score mean 0.09 (95% CI 0.12 to 0.06) at 12 mo; another study maintained ↓0.13 (95% CI 0.20 to 0.06) at 24 mo	Some studies show within-group reductions; between-group differences often not significant
6	Chaplais et al. (2015), France	Multidisciplinary smartphone-based	2–3 months	Weekly SMS, emails, booster sessions	Researchers	BMI/BMI *z*-score	One study: BMI decreased by 0.04 kg/m^2^ (SD 0.07); *z*-score = 2.03	Only two RCTs; electronic contact is often unsuccessful for weight loss
7	Fowler et al. (2021), USA	Technology-based interventions (multi-modal)	1–24 months	Apps, web, exergames, video-chats, wearables, in-person	HCPs, trained facilitators, researchers	Effect size (d)	Pooled mean effect (*n* = 32) *d* = −0.13, *P* = .001 (small effect)	27/33 treatment studies (79%) did not find significant differences between arms
8	Hammersley et al. (2016), Australia	Parent-focused eHealth	8 wks-2 yrs	Internet programs, IVR, telemedicine ± face-to-face	Counselors/HCPs	BMI/BMI *z*-score (SMD)	Meta-analysis SMD = −0.15 (95% CI −0.45 to 0.16), Z = 0.94, *P* = .35 (no significant effect)	Improved dietary measures in some studies; little PA improvement
9	Islam et al. (2020), Taiwan	mHealth (mobile apps)	6 weeks-9 months	Mobile apps ± emails, SMS, forums, counseling	Researchers/HCPs	BMI	Overall pooled change in BMI = −0.45 kg/m^2^ (95% CI −0.78 to −0.12), *P* = .008	Promising BMI reduction; PA effects less clear
10	Kaakinen et al. (2017), Finland	Technology-based counseling	4 weeks-2 years	Internet programs, multimedia games, SMS, CD-ROMs, calls	HCPs, researchers, educators	BMI	Four studies reported significantly lower BMI in intervention vs control (*P* < .001)	Improvements in fruit/veg consumption; mixed PA results
11	Kepper et al. (2021), USA	mHealth/Health IT	≤12 months (some longer follow-up)	Telehealth, CDS tools, apps, websites, SMS, email	Physicians, dietitians, nurses, health coaches	BMI/BMI *z*-score	Some studies: ↓0.12 units in BMI *z*-score and ↓0.49 units in BMI; others non-significant	Improved screening/documentation and referrals
12	Kouvari et al. (2022), Greece	Technology-based interventions	3–24 months	Mobile apps, web platforms, SMS, telemedicine ± face-to-face	Dietitians, physicians, psychologists	BMI (SMD)	Overall SMD = −0.61 (95% CI −1.10 to −0.13), *P* = .01; 6-mo SMD = −0.37 (95% CI −0.72 to −0.03), *P* = .03; 24-mo SMD = −0.31 (95% CI −0.63 to 0.02), *P* = .07	Parental involvement is important; effect reduced without parent involvement
13	Lam et al. (2022), USA	IoT-enabled eHealth	1 week to 9 months	Smartphone apps, smartwatches, NFC tags, smart garments	HCPs, families, schools	BMI	One study: average BMI decrease of 0.51 in overweight participants (no pooled estimate)	Adherence low; game-based IoT showed better effectiveness
14	Langarizadeh et al. (2021), Iran	Multicomponent mobile interventions	3 weeks to 6 months (varying)	Apps, SMS, phone sessions, face-to-face, web platforms	HCPs, researchers, automated systems	BMI/%BMIp95/zBMI	Some studies reported significant decreases in BMI, zBMI and %BMIp95 over 6 months	Mobile apps had mixed effects; some improvements in %BMIp95
15	Margetin et al. (2021), USA	Telehealth weight management	6–24 months	Web platforms, apps, email ± in-person sessions	HCPs including obesity specialists	BMI *z*-score	Random effects meta-analysis (10 RCTs) pooled net change in BMI *z*-score = 0.04 (95% CI −0.07 to 0.00), I2 = 12% (small effect)	No sig differences for BMI percentile or waist measures
16	Meidani et al. (2018), Iran	Phone-based/mHealth interventions (young children)	14 weeks to 5 years	Phone calls, in-person visits, educational materials, group sessions	Researchers/HCPs	Weight/BMI	Mixed results: 2 studies significant improvements in weight/BMI; 3 studies not significant	Mixed evidence for phone-based control in <6 yrs
17	Metzendorf et al. (2024), USA	mHealth smartphone interventions	2–24 months (median 3–24)	Smartphone apps, coaching, dietitians, PA coaches	Health professionals/coaches	BMI *z*-score/BMI	Median BMI *z*-score in adolescents ranged 2.2–2.5; small, generally non-sustained effects on BMI and PA	Small, transient improvements in diet/PA/QoL
18	Park et al. (2021), S. Korea	Child-centered ICT interventions	∼12 weeks (some longer)	Web platforms, apps, SMS, telehealth	Dietitians, nutritionists, researchers	BMI (WMD)	Overall WMD = −0.52 kg/m^2^ (95% CI −1.17 to 0.13) (not significant); Web interventions WMD = −1.26 kg/m^2^ (95% CI −2.24 to −0.28) (significant)	Lifestyle comparator and obesity-targeted interventions showed larger effects
19	Partridge et al. (2020), Australia	Multicomponent (text + apps + wearables + in-person)	2–24 months	Text messages, web modules, wearable devices, in-person sessions	Health educators, dietitians, clinical staff	BMI	Reported mean BMI across studies ∼29.7 (SD 1.6); varied intervention-specific results	Heterogeneous interventions; some with wearables/apps showed changes
20	Quelly et al. (2016), USA	mHealth mobile apps	9 days to 20 weeks	Smartphone/tablet apps for self-monitoring, goal setting	Researchers/HCPs	BMI	One study: mean BMI decrease 0.51 in overweight/obese participants; others no significant BMI change	Apps improved motivation, goal setting, some dietary habits
21	Qiu et al. (2022), China	eHealth lifestyle interventions	6 weeks to 24 months	Mobile apps, web platforms, telehealth, active video games	Researchers/HCPs	BMI (WMD) and BMI *z*-score	WMD for BMI = −0.32 (95% CI −0.50 to −0.13); BMI *z*-score WMD = −0.08 (95% CI −0.14 to −0.03)	eHealth more effective for BMI, weight; high heterogeneity (I2)
22	Shin et al. (2019), Korea	mHealth (text, apps, phone)	8–72 weeks	Text messaging, apps, phone calls, combined with clinic visits	Health professionals/researchers	BMI (SMD)	Pooled (3 studies) SMD = −0.043 (95% CI −0.322 to 0.236) (NS); other meta-analysis SMD = 0.341 (95% CI 0.019 to 0.664) (significant depending on subgroup)	Short-term and text-only interventions showed larger effects
23	Smith et al. (2013), USA	eHealth (EHRs, telemedicine, text)	Varied (some 1 year)	EHRs, telemedicine, text/phone support	Pediatricians, dietitians, counselors	BMI/ Screening odds	Odds of BMI screening higher with EHRs: OR = 2.53 (95% CI 1.39–4.62); telemedicine similar BMI percentile changes to in-person	Improved access and screening; mixed-weight outcomes
24	Turner et al. (2015), USA	mHealth (SMS, apps, wearables)	2 weeks to 12 months	SMS, apps, games, PDAs, wearable sensors	HCPs, researchers	BMI SDS/BMI	One study: non-significant decrease in BMI-SDS 0.07 ± 0.26 kg/m^2^ after 9 months (*P* < .1)	Combination of SMS with other treatments did not add effect
25	Wang et al. (2022), China	Wearable devices	2.5–18 months	Wearables with self-monitoring, goal setting, feedback	Researchers/HCPs/school staff	BMI and BMI *z*-score (MD)	MD in BMI = −0.23 (95% CI −0.43 to −0.03), *P* = .03; BMI *z* MD = −0.07 (95% CI −0.13 to −0.01), *P* = .01	Wearables showed small but significant BMI reductions
26	Yau et al. (2022), Canada	mHealth apps (prevention)	≤1 month to 8 months	Mobile apps with gamification, diaries, wearables, SMS	Researchers/HCPs	BMI/%BMI per day	Significant decrease in %BMI from baseline to end (baseline mean −0.051, SD 0.013%BMI/day, *P* < .01); clinic setting: coefficient = −0.02 (95% CI −0.03 to −0.01, *P* < .001)	Improvements in PA (4/8), diet (5/6); BMI significant in 2/6 studies
27	Wang et al. (2024), China	mHealth app-based interventions	2–48 weeks	Smartphone-based mHealth apps	Researchers/HCPs	Physical activity (SMD) and BMI outcomes	mHealth increased total PA SMD = 0.29 (95% CI 0.13–0.45); MVPA SMD = 0.11 (95% CI −0.04 to 0.25) (NS)	PA increased; BMI effects inconsistent

## Details of interventions

Across the 27 included systematic reviews, DHIs aimed at preventing or managing childhood obesity demonstrated considerable variation in their design, delivery, setting, duration, interactivity, and behavioral targets.

## Providers of the interventions

Intervention providers varied widely across the included reviews. Many interventions were delivered by healthcare professionals, including pediatricians, dietitians, nurses, psychologists, and other trained staff.^21,22,31,32^ Several school-based interventions involved teachers or trained facilitators.^23,33^ In some cases, interventions were entirely automated or self-guided, with no facilitator.^29^ Parent or caregiver involvement was a key component in family-based programs.^16,19^

## Delivery methods

DHIs included in the 27 systematic reviews were delivered using a broad range of digital technologies tailored to both prevention and management of childhood obesity. The most commonly reported platforms were mobile applications, used either as standalone tools or in combination with other modalities, to support behavior change through goal setting, feedback, and self-monitoring.^15,16,34^ Several interventions incorporated SMS or text messaging to deliver reminders, motivational content, or progress updates, particularly in programs focusing on sustained engagement and adherence.^21,31,35,36^

Wearable activity trackers were often integrated with mobile apps or web platforms to provide real-time monitoring of physical activity, particularly in interventions aimed at increasing step count or reducing sedentary time.^27,37^ Web-based platforms and educational portals were commonly used in school-based and family-focused interventions, offering structured modules, gamified learning, or interactive content.^23,26,33,38,39^ A subset of reviews described telehealth, video consultations, and remote monitoring systems, especially in management-oriented interventions where clinical supervision or behavioral counseling was needed.^18,22,40^

## Settings

Home-based interventions were delivered in prevention and management contexts, allowing children, adolescents, and families to interact with mobile apps, SMS programs, and online tools within their daily routines.^16,20,24,25,31,34–36^^,38^

School-based settings were considered for prevention-focused interventions, where digital programs were embedded into curricula or implemented in coordination with educators. These included structured online learning, gamified content, and wearable tracking, including home and school elements.^16,23,26,33,38,39^

Clinical settings were used primarily for management interventions targeting overweight or children with obesity. These often include telehealth consultations, remote monitoring, or integration with healthcare provider visits.^15,17,18,21,22,34,40^

Several reviews featured mixed or community-based settings, combining digital support across home, school, and clinical environments or through broader public health or family systems. For instance, exergames and PA interventions were provided in home and school contexts.^29^ Reviews described digital tools that were applicable in a range of contexts and adaptable to multiple delivery points.^19,30,37,41^

## Duration and intensity

Several short-term interventions lasted for 4–12 weeks, especially those focused on initial behavior change, feasibility testing, or gamified activities.^17,19,29^ Other reviews included programs with medium-term durations between 3 and 6 months,^20,36^ while many management-focused interventions extended to 12 months or longer to support sustained weight control and follow-up.^18,22,31,40^

Intensity interventions included daily self-monitoring or app usage, weekly coaching or SMS reminders, or monthly counseling or feedback sessions. Programs involving continuous app engagement and frequent behavioral change spread between 2 and 12 months.^16,27^ Reviews noted that some studies used multiple follow-up points and integrated feedback cycles to enhance adherence.^30,39^ Multicomponent interventions often had higher intensity due to combinations of app use, teleconsultation, in-person sessions, and behavioral reinforcement.^21,24^

## Prevention-focused interventions

Nine of the included systematic reviews primarily focused on preventing obesity among children and adolescents who were not necessarily overweight or children with obesity at baseline. These reviews assessed the effectiveness of digital health interventions (DHIs) that encourage healthy behaviors such as improved dietary habits, increased physical activity, and reduced sedentary time. Interventions were delivered through mobile health applications, SMS-based reminders, wearable devices, and school-based eHealth platforms. Reviews reported improvements in health behaviors, motivation, and knowledge. However, the effects on anthropometric outcomes such as BMI were generally modest or inconsistently reported, particularly in short-term interventions or those without personalized feedback components.^16,20,23,26,28,33,37–39^

## Management-focused interventions

Eleven reviews specifically targeted the management of overweight or obesity in children and adolescents already identified as having excess weight. These interventions aimed to reduce BMI, BMI *z*-scores, or improve weight-related health behaviors through structured and often personalized digital approaches. Common modalities included telehealth consultations, self-monitoring apps, SMS follow-up systems, and interactive platforms with behavior change techniques. Reviews found that interventions involving parental support, personalized goal-setting, and regular feedback yielded better adherence and modest but meaningful reductions in weight-related outcomes. Longer-duration and family-engaged interventions were generally more effective in sustaining improvements.^15,17,18,21,22,31,34–36^^,40^

## Combined prevention and management interventions

Seven reviews addressed prevention and management objectives or included populations with mixed weight status without clearly delineating between preventive and therapeutic goals. These reviews evaluated various digital tools, including mobile apps, exergames, web-based interventions, and virtual coaching, which were delivered in various settings. Few reviews included interventions applied to healthy-weight and overweight children, aiming to improve behaviors such as physical activity, healthy eating, and digital self-monitoring. While many of these reviews noted positive engagement and behavioral improvements, the diversity in populations and intervention targets made it difficult to draw consistent conclusions regarding weight-related outcomes.^19,24,25,29,30,41^

## Discussion

The global increase in childhood obesity presents a substantial public health challenge, demanding innovative and scalable interventions.^42^ This overview of systematic reviews highlights the emerging potential of DHIs in addressing this issue among children and adolescents. The results recommend that DHIs, ranging from mobile applications and SMS based programs to web platforms, wearable devices, and telehealth, can positively impact weight-related outcomes and health behaviors of the pediatric population.

The systematic reviews included in this overview included various strategies for preventing and managing childhood obesity. Web-based interventions have been viewed due to their accessibility, scalability, and ability to reach a large audience.^22,23,29,33^ These interventions typically involve educational resources, interactive tools, and behavioral support mechanisms utilized by websites or online platforms. Although the reviews suggest a moderate success in reducing obesity-related metrics, the effectiveness of web-based interventions often differs depending on the intervention's design, duration, and target population. Furthermore, mHealth interventions, which utilize mobile applications and smartphones, demonstrated promising results. Reviews on mHealth interventions indicate that these solutions can effectively provide real-time feedback, promote behavioral change, and promote physical activity or dietary adjustments..^16,20,26,27,30,35,38–41^ Mobile health technologies, particularly when combined with personalized feedback, provide an opportunity to engage children and adolescents in managing their health outside of clinical settings. Another review supports these findings, which identified that mobile applications have increased the adoption of healthy eating behaviors among children and adolescents with obesity.^43^ Throughout the included reviews, several cross-cutting themes emerged that shape the effectiveness of DHIs. Personalization was consistently linked to improved engagement and outcomes, particularly in mHealth interventions that provided tailored feedback. However, the findings of similar interventions were sometimes ambiguous due to differences in population characteristics, intervention intensity, and outcome measures. Similarly, while some mHealth reviews reported improvements in physical activity and diet, others showed minimal impact, possibly indicating shorter durations or lack of customization. However, there is still considerable flexibility in the long-term effectiveness of these interventions, and factors such as user engagement and app usability must be considered when evaluating the outcome.

Parental involvement also appeared critical; interventions that included caregivers tended to demonstrate greater adherence and sustained effects, while those targeting \children alone struggled with long-term engagement. The setting of the intervention, whether clinical, school-based, or home-based, was also influenced by outcomes, with structured environments providing consistency and home-based programs providing flexibility but facing difficulties in supervision.

The use of multicomponent interventions, which combine multiple digital platforms such as mobile apps, text messages, phone sessions, face-to-face meetings, and web-based platforms, was considered adequate.^17,24,36^ These interventions possess the most significant potential for success due to their comprehensive and holistic approach to obesity management. These interventions address various aspects of behavior change, including physical activity, dietary habits, and psychological factors. These results are supported by Monninghoff et al., who reported that mHealth interventions significantly affected obesity prevention in youth, particularly regarding physical activity levels.^43^ The combination of digital tools and human interaction may enhance user involvement and adherence to the intervention, making it a valuable strategy for tackling childhood obesity. Digital interventions have demonstrated significant improvements in child BMI and a substantial impact on diet and physical activity, with emerging evidence indicating the use of social media and video gaming to enhance programming.^9^ Multicomponent interventions that combined digital tools with human interaction were generally more effective than standalone ones. These comprehensive strategies addressed multiple behavioral domains and enhanced user engagement. Despite promising results, equity concerns persist, as limited access to devices, the internet, and digital literacy may hinder the ability of DHIs to reach low-resource settings.

In addition to the available intervention, one review on the use of wearable devices was conducted as part of the obesity management strategy.^28^ Wearables like fitness trackers and smartwatches can monitor physical activity, sleep patterns, and even caloric intake, providing users with continuous feedback. These devices may contribute to behavior change by providing objective data and promoting accountability. These findings are supported by a review that reports that wearable activity tracking devices can lead to behavioral changes in children, leading to increased physical activity, resulting in the prevention of pediatric obesity.^44^ The reviews also highlighted the role of telehealth technology in managing obesity. While only three reviews focused on this approach, telehealth interventions, which offer remote consultations and support, can alleviate the issue for children and families with limited access to traditional healthcare services.^18,21,31^ These interventions provide a flexible and convenient approach for healthcare professionals to offer tailored obesity management strategies, including counseling and lifestyle modifications. These findings align with a review by Moorman et al., who reported that pediatric telehealth weight management interventions demonstrate practical feasibility and acceptance and address time, travel, and cost barriers to in-person interventions, thus enhancing accessibility.^45^

Despite these promising outcomes, several challenges persist. Sustainability of the user engagement remains a major barrier, particularly among children and adolescents. Interventions that do not involve caregivers may find it difficult to maintain long-term adherence. Additionally, the digital device poses a significant equity concern. Limited access to smartphones, internet connectivity, or digital literacy can hinder the effectiveness and reach of DHIs, especially in low-resource settings. Although some reviews included meta-analyses indicating modest reductions in BMI *z*-scores and percentiles, we did not obtain data due to the heterogeneity in study designs, populations, and outcome measures. Other anthropometric outcomes such as waist circumference, body fat percentage, and skinfold thickness were reported to be inconsistent, further limiting synthesis.

The quality of the included reviews varied from low to moderate, with many showing design limitations and inconsistent reporting. These findings suggest that DHIs possess promise, but require thoughtful design, contextual adaptation, and integration into more comprehensive public health strategies. Developers should focus on personalization and usability. Physicians should consider combining digital tools with human assistance. Policymakers must address infrastructure gaps to ensure equitable access to infrastructure. High-quality trials with standardized outcomes and long-term follow-up are essential to enhance the evidence base and guide implementation.

## Conclusion

This overview of systematic reviews highlights the importance of DHI's in addressing childhood obesity. Mobile app-based and SMS-based interventions consistently established modest but meaningful improvements in BMI and health-related behaviors. Web-based and multicomponent interventions also exhibited promising results, especially when integrated into school or family settings. Wearable devices and telehealth platforms further expanded the reach and adaptability of obesity management strategies. However, ongoing research is necessary to refine these interventions, address barriers to participation, and assess their long-term effectiveness. Future research should focus on identifying multidisciplinary approaches for developing digital tools, including parental involvement, and ensuring equitable access to these interventions to maximize their impact on childhood obesity prevention and management.

The findings of this overview recommend the integration of DHIs into broader public health interventions for the prevention and management of childhood obesity. Policymakers and practitioners should prioritize interventions that are evidence-based, user-friendly, and adaptable to various contexts. Emphasizing family involvement and access to digital technology is crucial for expanding the impact of digital solutions. This review lays a foundation for designing effective and scalable digital interventional strategies to prevent and manage childhood obesity across diverse settings.

## Supplemental Material

sj-docx-1-dhj-10.1177_20552076261415914 - Supplemental material for Digital health interventions for childhood obesity: An umbrella review Supplemental material, sj-docx-1-dhj-10.1177_20552076261415914 for Digital health interventions for childhood obesity: An umbrella review by Edlin Glane Mathias, Siva N, Elstin Anburaj S, Vaishnavi Naik and Baby S Nayak in DIGITAL HEALTH

sj-docx-2-dhj-10.1177_20552076261415914 - Supplemental material for Digital health interventions for childhood obesity: An umbrella review Supplemental material, sj-docx-2-dhj-10.1177_20552076261415914 for Digital health interventions for childhood obesity: An umbrella review by Edlin Glane Mathias, Siva N, Elstin Anburaj S, Vaishnavi Naik and Baby S Nayak in DIGITAL HEALTH

sj-docx-3-dhj-10.1177_20552076261415914 - Supplemental material for Digital health interventions for childhood obesity: An umbrella review Supplemental material, sj-docx-3-dhj-10.1177_20552076261415914 for Digital health interventions for childhood obesity: An umbrella review by Edlin Glane Mathias, Siva N, Elstin Anburaj S, Vaishnavi Naik and Baby S Nayak in DIGITAL HEALTH
