# Supplementary material for: Digital health interventions for childhood obesity: An umbrella review
Source: Digit Health. 2026 Apr 1;12:20552076261415914. doi: 10.1177/20552076261415914 (PMC13049355; doi:10.1177/20552076261415914)
Supplement: sj-docx-1-dhj-10.1177_20552076261415914 - Supplemental material for Digital health interventions for childhood obesity: An umbrella review [file sj-docx-1-dhj-10.1177_20552076261415914.docx]

**Appendix-1 (Search strategy)**

**Digital Health Interventions**

"digital health"[MeSH Terms] OR "digital health" [Text Word] OR "Digital Health Technology" [Text Word] OR "ehealth"[Text Word] OR "e-health"[Text Word] OR "mobile health"[Text Word] OR "digital medicine"[Text Word] OR "digital technology"[Text Word] OR "electronic health"[Text Word] OR "telehealth"[Text Word] OR "mhealth"[Text Word] OR "Smartphone"[Text Word] OR "telecare"[Text Word] OR "m-health"[Text Word] OR "Telemedicine"[Text Word] OR "cellphone*"[Text Word] OR "smartphone*"[Text Word] OR "remote consultation"[MeSH Terms] OR "remote consultation"[Text Word] OR "internet based intervention"[MeSH Terms] OR "internet based intervention"[Text Word] OR "web based intervention"[Text Word] OR "mobile applications"[MeSH Terms] OR "mobile applications"[Text Word] OR "cell phone use"[MeSH Terms] OR "cell phone use"[Text Word] OR "health information technology"[Text Word] OR "Telemedicine"[MeSH Terms] OR "Telemedicine"[Text Word]

**Children**

"child"[MeSH Terms] OR "children"[Text Word] OR "Child"[Text Word] OR "teen*"[Text Word] OR "Adolescent"[Text Word] OR "Child"[Text Word] OR "adolescen*"[Text Word]

**Obesity**

"obesity"[MeSH Terms] OR "obesity"[Text Word] OR "body weight"[MeSH Terms] OR "body weight"[Text Word] OR "obes*"[Text Word] OR "overweight"[MeSH Terms] OR "overweight"[Text Word] OR "overweight"[Text Word] OR "pediatric obesity"[MeSH Terms] OR "pediatric obesity"[Text Word] OR "adolescent obesity"[Text Word] OR "obesity, abdominal"[MeSH Terms] OR "abdominal obesity"[Text Word] OR "child obesity"[Text Word] OR "body mass index"[MeSH Terms] OR "body mass index"[Text Word] OR "BMI"[Text Word] OR "obesity, morbid"[MeSH Terms] OR "morbid obesity"[Text Word] OR "skinfold thickness"[MeSH Terms] OR "skinfold thickness"[Text Word] OR "adiposity"[MeSH Terms] OR "adiposity"[Text Word] OR "body weight changes"[MeSH Terms] OR "body weight changes"[Text Word] OR "weight change"[Text Word] OR "weight loss"[MeSH Terms] OR "weight loss"[Text Word] OR "weight reduction"[Text Word]

**Systematic review filter**

(((systematic review[ti] OR systematic literature review[ti] OR systematic scoping review[ti] OR systematic narrative review[ti] OR systematic qualitative review[ti] OR systematic evidence review[ti] OR systematic quantitative review[ti] OR systematic meta-review[ti] OR systematic critical review[ti] OR systematic mixed studies review[ti] OR systematic mapping review[ti] OR systematic cochrane review[ti] OR systematic search and review[ti] OR systematic integrative review[ti]) NOT comment[pt] NOT (protocol[ti] OR protocols[ti])) NOT MEDLINE [subset]) OR (Cochrane Database Syst Rev[ta] AND review[pt]) OR systematic review[pt]

| **Author name & year** | **1** | **2** | **3** | **4** | **5**  **Appendix 2 (Methodological quality assessment)** | **6** | **7** | **8** | **9** | **10** | **11** | **12** | **13** | **14** | **15** | **16** | **Rating overall confidence** |
| --- | --- | --- | --- | --- | --- | --- | --- | --- | --- | --- | --- | --- | --- | --- | --- | --- | --- |
| Amminudin et al., 2019 | Yes | Unclear | Yes | Yes | Yes | Yes | No | Yes | Unclear | Unclear | Yes | NA | NA | NR | No | NA | Critically Low |
| Antwi et al., 2013 | Yes | Yes | Yes | Yes | Yes | Yes | No | Yes | Yes | Yes | Yes | NA | NA | NR | No | NA | moderate |
| Azevedo et al., 2021 | Yes | Yes | Yes | Yes | Yes | Yes | No | Yes | Yes | Yes | Yes | Yes | Yes | Yes | Yes | Yes | moderate |
| Bonvicini et al., 2022 | Yes | Yes | Yes | Yes | Yes | Yes | No | Yes | Yes | Yes | Yes | Yes | Yes | NR | Yes | NA | moderate |
| Catherine et al., 2015 | Yes | Yes | Yes | Yes | Yes | Yes | No | Yes | Yes | Yes | Yes | NR | NR | NR | Yes | NR | moderate |
| Chaplais et al., 2015 | Yes | Yes | Yes | Yes | Yes | Yes | No | Yes | Yes | Yes | Yes | Yes | NR | NR | Yes | NR | moderate |
| Fowler et al., 2021 | Yes | Yes | Yes | Yes | Yes | Yes | No | Yes | Yes | Yes | Yes | Yes | Yes | Yes | Yes | NR | moderate |
| Hammersley et al., 2016 | Yes | Yes | Yes | Yes | Yes | Yes | No | Yes | Yes | Yes | Yes | Yes | Yes | Yes | Yes | NR | moderate |
| Islam et al., 2020 | Yes | Yes | Yes | Yes | Yes | Yes | No | Yes | Yes | Yes | Yes | Yes | Yes | Yes | Yes | NR | moderate |
| Kaakinen et al., 2017 | Yes | Yes | Yes | Yes | Yes | Yes | No | Yes | Yes | Yes | Yes | NR | NR | NR | Yes | NR | moderate |
| Kepper et al., 2021 | Yes | Yes | Yes | Yes | Yes | Yes | No | Yes | Yes | Yes | Yes | NR | NR | NR | Yes | NR | moderate |
| Kouvari et al., 2022 | Yes | Yes | Yes | Yes | Yes | Yes | No | Yes | Yes | Yes | Yes | Yes | Yes | Yes | Yes | Yes | moderate |
| Lam et al., 2022 | Yes | Not reported | Yes | Yes | Yes | Yes | No | Yes | Yes | Yes | Yes | NR | NR | NR | Yes | Yes | Low |
| Langarizadeh et al., 2021 | Yes | Yes | Yes | Yes | Yes | Yes | No | Yes | Yes | Yes | Yes | NR | NR | NR | Yes | Yes | moderate |
| Margetin et al., 2021 | Yes | Yes | Yes | Yes | Yes | Yes | No | Yes | Yes | Yes | Yes | Yes | NA | Yes | Yes | Yes | moderate |
| Meidani et al., 2018 | Yes | Yes | Yes | Yes | Yes | Yes | No | Yes | Yes | Yes | Yes | NA | NA | NA | Yes | Yes | moderate |
| Metzendorf et al., 2024 | Yes | Yes | Yes | Yes | Yes | Yes | Yes | Yes | Yes | Yes | Yes | NA | NA | NA | Yes | Yes | moderate |
| Park et al., 2021 | Yes | Yes | Yes | Yes | Yes | Yes | No | Yes | Yes | Yes | Yes | Yes | Yes | Yes | Yes | Yes | moderate |
| Partridge et al., 2020 | Yes | Yes | Yes | Yes | Yes | Yes | No | Yes | Yes | Yes | Yes | Yes | Yes | Yes | Yes | Yes | moderate |
| Quelly et al., 2016 | No | Unclear | Yes | Unclear | Unclear | Unclear | No | Yes | Unclear | Unclear | Yes | NA | NA | NA | Unclear | NA | Critically Low |
| Qiu et al., 2022 | Yes | Yes | Yes | Yes | Yes | Yes | No | Yes | Yes | Yes | Yes | Yes | Yes | Yes | Yes | Yes | moderate |
| Shin et al., 2019 | Yes | Yes | Yes | Yes | Yes | Yes | No | Yes | Yes | Yes | Yes | Yes | Yes | Yes | Yes | Yes | moderate |
| Smith et al., 2013 | Yes | Yes | Yes | Yes | Yes | Yes | No | Yes | Yes | Yes | Yes | NA | NA | NA | Yes | NA | moderate |
| Turner et al., 2015 | Unclear | Yes | Yes | Yes | Yes | Yes | No | Yes | Yes | Yes | Yes | NA | NA | NA | Yes | NA | moderate |
| Wang et al., 2022 | Yes | Yes | Yes | Yes | Yes | Yes | No | Yes | Yes | Yes | Yes | Yes | Yes | Yes | Yes | Yes | moderate |
| Yau et al., 2022 | Yes | Yes | Yes | Yes | Yes | Yes | No | Yes | Yes | Yes | Yes | NA | NA | NA | Yes | NA | moderate |
| Wang et al., 2024 | Yes | Yes | Yes | Yes | Yes | Yes | No | Yes | Yes | Yes | Yes | Yes | Yes | Yes | Yes | Yes | moderate |

NA: Not Applicable; NR: Not reported
