# Supplementary material for: Digital health interventions for childhood obesity: An umbrella review
Source: Digit Health. 2026 Apr 1;12:20552076261415914. doi: 10.1177/20552076261415914 (PMC13049355; doi:10.1177/20552076261415914)
Supplement: sj-docx-3-dhj-10.1177_20552076261415914 - Supplemental material for Digital health interventions for childhood obesity: An umbrella review [file sj-docx-3-dhj-10.1177_20552076261415914.docx]

# Preferred Reporting Items for Resistance Exercise Studies (PRIRES)

Note: This review did not primarily evaluate resistance exercise interventions. Therefore, several PRIRES items related specifically to resistance training prescription and delivery are marked as Not Applicable (NA) with justification, which is methodologically appropriate.

| Item No. | PRIRES Reporting Item | Reported (Yes / No / NA) | Location in Manuscript / Justification |
| --- | --- | --- | --- |
| 1 | Identification of resistance exercise study in title | NA | Umbrella review of digital health interventions |
| 2 | Clear description of study design | Yes | Methods – Study design |
| 3 | Population characteristics clearly reported | Yes | Methods – Eligibility criteria |
| 4 | Sample size justification | NA | Secondary synthesis; not applicable |
| 5 | Eligibility criteria clearly defined | Yes | Methods – Eligibility criteria |
| 6 | Description of resistance exercise modality | NA | Resistance exercise not primary intervention |
| 7 | Exercise equipment described | NA | Not applicable |
| 8 | Exercise intensity defined (e.g., %1RM, RPE) | NA | Not applicable |
| 9 | Volume of exercise (sets, reps) | NA | Not applicable |
| 10 | Exercise frequency | NA | Not applicable |
| 11 | Session duration | NA | Not applicable |
| 12 | Intervention duration | NA | Not applicable |
| 13 | Progression model described | NA | Not applicable |
| 14 | Rest intervals between sets | NA | Not applicable |
| 15 | Supervision level described | NA | Not applicable |
| 16 | Qualifications of supervisors | NA | Not applicable |
| 17 | Adherence/compliance assessment | Partial | Narrative synthesis of engagement |
| 18 | Strategies to improve adherence | Partial | Results – Intervention characteristics |
| 19 | Comparator/control condition described | Yes | Methods – Comparator |
| 20 | Primary outcomes clearly defined | Yes | Methods – Outcomes |
| 21 | Secondary outcomes defined | Yes | Methods – Outcomes |
| 22 | Resistance-specific outcomes (strength, power) | NA | Not assessed |
| 23 | Outcome measurement tools described | Yes | Methods – Outcomes |
| 24 | Timing of outcome assessment | Yes | Tables 1 and 2 |
| 25 | Adverse events reporting | NA | Not reported in included reviews |
| 26 | Risk of bias/quality assessment | Yes | Methods – AMSTAR-2 |
| 27 | Data synthesis methods | Yes | Methods – Data synthesis |
| 28 | Heterogeneity assessment | Yes | Results – Narrative synthesis |
| 29 | Subgroup analysis | NA | Not performed |
| 30 | Sensitivity analysis | NA | Not performed |
| 31 | Flow of studies through review | Yes | PRISMA flow diagram (Figure 1) |
| 32 | Characteristics of included studies | Yes | Table 1 |
| 33 | Description of interventions | Yes | Table 2 |
| 34 | Consistency of findings across studies | Yes | Results |
| 35 | Discussion of clinical relevance | Yes | Discussion |
| 36 | Interpretation in context of existing literature | Yes | Discussion |
| 37 | Limitations related to intervention reporting | Yes | Discussion – Limitations |
| 38 | Implications for practice | Yes | Discussion |
| 39 | Implications for future resistance exercise research | Partial | Discussion – Future research |
| 40 | Protocol registration | Yes | PROSPERO |
| 41 | Funding sources | Yes | Funding section |
| 42 | Conflicts of interest | Yes | Conflicts of interest |
| 43 | Data availability | Partial | Supplementary files |
